# Association between Pericytes in Intraplaque Neovessels and Magnetic Resonance Angiography Findings

**DOI:** 10.3390/ijms21061980

**Published:** 2020-03-13

**Authors:** Atsushi Ogata, Tomihiro Wakamiya, Masashi Nishihara, Tatsuya Tanaka, Taichiro Mizokami, Jun Masuoka, Nobuaki Momozaki, Shuji Sakata, Hiroyuki Irie, Tatsuya Abe

**Affiliations:** 1 Department of Neurosurgery, Faculty of Medicine, Saga University, Saga 840-8501, Japan; wakamiya@cc.saga-u.ac.jp (T.W.); s96047@hotmail.com (T.T.); mizmiztaich@gmail.com (T.M.); masuoka@cc.saga-u.ac.jp (J.M.); abet@cc.saga-u.ac.jp (T.A.); 2 Department of Radiology, Faculty of Medicine, Saga University, Saga 840-8501, Japan; nishiham@cc.saga-u.ac.jp (M.N.); irie@cc.saga-u.ac.jp (H.I.); 3 Department of Neurosurgery, Imari Arita Kyoritsu Hospital, Imari 849-4193, Japan; momozaki-n@imari-arita-hp.or.jp; 4 Department of Neurosurgery, Saga Ken Medical Center Koseikan, Saga 840-8571, Japan; ssakata3@gmail.com

**Keywords:** carotid artery, carotid stenosis, pericyte, magnetic resonance imaging

## Abstract

(1) Background: Pericytes are involved in intraplaque neovascularization of advanced and complicated atherosclerotic lesions. However, the role of pericytes in human carotid plaques is unclear. An unstable carotid plaque that shows high-intensity signals on time-of-flight (TOF) magnetic resonance angiography (MRA) is often a cause of ischemic stroke. The aim of the present study is to examine the relationship between the pericytes in intraplaque neovessels and MRA findings. (2) Methods: A total of 46 patients with 49 carotid artery stenoses who underwent carotid endarterectomy at our hospitals were enrolled. The patients with carotid plaques that were histopathologically evaluated were retrospectively analyzed. Intraplaque hemorrhage was evaluated using glycophorin A staining, and intraplaque neovessels were evaluated using CD34 (Cluster of differentiation) stain as an endothelial cell marker or NG2 (Neuron-glial antigen 2) and CD146 stains as pericyte markers. Additionally, the relationships between the TOF-MRA findings and the carotid plaque pathologies were evaluated. (3) Results: Of the 49 stenoses, 28 had high-intensity signals (TOF-HIS group) and 21 had iso-intensity signals (TOF-IIS group) on TOF-MRA. The density of the CD34-positive neovessels was equivalent in both groups. However, the NG2- and CD146-positive neovessels had significantly higher densities in the TOF-HIS group than in the TOF-IIS group. (4) Conclusion: The presence of a high-intensity signal on TOF-MRA in carotid plaques was associated with intraplaque hemorrhage and few pericytes in intraplaque neovessels. These findings may contribute to the development of new therapeutic strategies focusing on pericytes.

## 1. Introduction

An unstable carotid plaque can cause thromboembolic stroke [1,2]. Unstable carotid plaques are defined as plaques with intraplaque hemorrhage (IPH), an incomplete fibrous cap, and a large lipid-rich necrotic core [3]. In particular, intraplaque neovessel formation and plaque rupture with IPH have been shown to be highly associated with adverse events [4,5]. In a previous report, neovessel density was not associated with the presence or absence of IPH [6].

The association between IPH and the vulnerability of intraplaque neovessels has not yet been studied. We have focused on pericytes of intraplaque neovessels and reported that pericytes may be involved in carotid plaque hemorrhage based on evaluations of plaque from patients who underwent carotid endarterectomy (CEA) [7]. Besides this, a histopathological study using anti-NG2 and CD146 antibodies as pericyte markers showed that pericytes in carotid plaque were associated with asymptomatic carotid stenosis [8]. The pericytes may play a role in stabilizing plaques in the carotid artery [8]. Endothelial cells are covered by pericytes in capillary vessels, and pericytes play a major role in capillary maturation and stabilization during angiogenesis [9]. However, the role of pericytes in the intraplaque neovessels prior to carotid endarterectomy and stenting is unclear. IPH has been reported as the principal factor for progression to unstable plaque and can be confirmed on magnetic resonance imaging (MRI) of human carotid plaques [10]. Additionally, an association between high-intensity carotid plaque on time-of-flight (TOF) magnetic resonance angiography (MRA) and IPH has been reported [11]. The present study aims to elucidate the associations between the pericytes in intraplaque neovessels and the MRA findings.

## 2. Results

This study assessed 46 patients (43 men and 3 women) with 49 carotid artery stenoses who underwent CEA with histopathological evaluation of the plaques. The average age of patients was 69 years (range, 38–87 years). There were 39 (80%) symptomatic lesions and 10 (20%) asymptomatic lesions. Overall, 36 (78%) had a history of hypertension, 29 (63%) had dyslipidemia, 22 (45%) had type 2 diabetes mellitus, 17 (35%) had coronary artery disease, and 28 (57%) had a history of smoking. A total of 11 patients (22%) underwent urgent CEA. Furthermore, 5 patients (10%) had a high intensity area in the ipsilateral brain on MRI-diffusion weighted imaging (DWI) after CEA. Restenosis after CEA occurred in 3 (6%) patients (Table 1).

**Figure 1:**
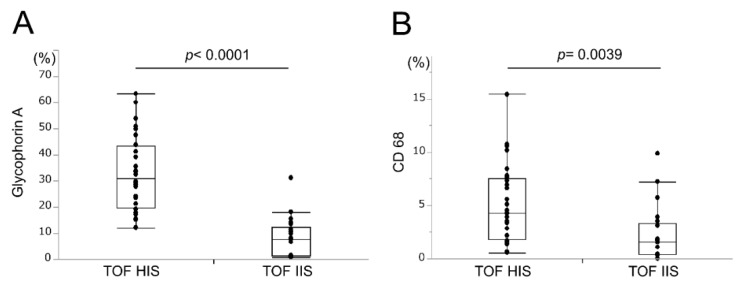
Relationships between time-of-flight magnetic resonance angiography (TOF-MRA) signal intensity and (**A**) glycophorin A-positive area and (**B**) CD68-positive area. The glycophorin A and CD68-positive areas are significantly larger in the TOF-HIS group (high-intensity signal plaques) than in the TOF-IIS (iso-intensity signal plaques) group. *p*-values are based on the Student’s t-test.

On TOF-MRA analysis, 28 lesions had high-intensity signal plaques (TOF-HIS group), and 21 had iso-intensity signal plaques (TOF-IIS group). The area that stained positive for glycophorin A was 32.8% ± 2.7% in the TOF-HIS group and 8.3% ± 1.7% in the TOF-IIS group (*p* < 0.0001; Figure 1A), and the percentage of CD68-positive stained area was 5.1% ± 0.7% in the TOF-HIS group and 2.2% ± 0.6% in the TOF-IIS group (*p* = 0.0039; Figure 1B).

**Figure 2:**
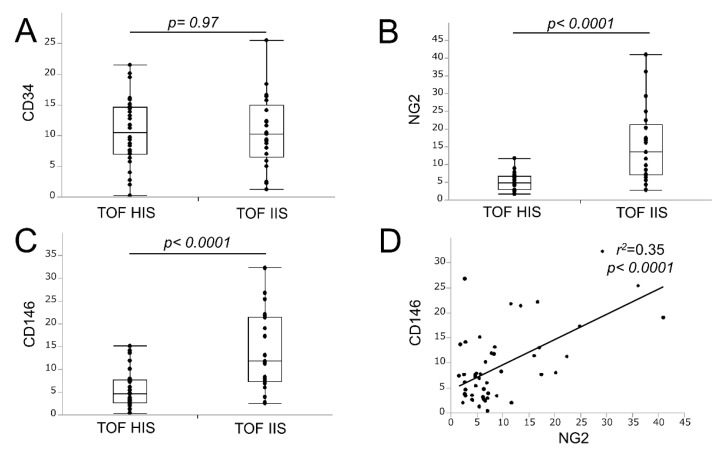
Relationship between TOF-MRA signal intensity and the number of neovessels. The difference in the number of CD34-positive neovessels according to TOF-MRA signal intensity is not significant. The *p*-value is based on the Student’s t-test (**A**). The numbers of NG2-positive (**B**) and CD146-positive (**C**) neovessels are significantly lower in the TOF-HIS group than in the TOF-IIS group. *P*-values are based on the Student’s t-test. The correlation between the numbers of NG2 and of CD146-positive neovessels is significant. Spearman’s rank correlation test was used to assess correlations (**D**).

There was no significant difference in the mean number of neovessels stained with CD34 between the groups (10.7 ± 1.0 vs. 10.6 ± 1.3; *p* = 0.97; Figure 2A), but the mean number of neovessels stained with NG2 was significantly higher in the TOF-IIS group than in the TOF-HIS group (5.0 ± 0.4 vs. 15.5 ± 2.3; *p* < 0.0001; Figure 2B). Furthermore, the TOF-HIS group had a significantly lower mean number of CD146-positive neovessels than the TOF-IIS group (5.9 ± 0.8 vs. 13.9 ± 1.9; *p* < 0.0001; Figure 2C). These statistical results are presented in Table 2. The results were similar when two observers analyzed the results independently, and there was low interobserver variability (Table 3). There was a significant correlation in the mean numbers between NG2 and CD146-positive neovessels (Figure 2D). However, there was no correlation between the mean number of NG2-positive neovessels and the CD68-positive area (r^2^ = 0.048; *p* = 0.13). In addition, there was no significant correlation between the mean number of CD146-positive neovessels and the CD68-positive area (*r*^2^ = 0.07, *p* = 0.07).

**Figure 3:**
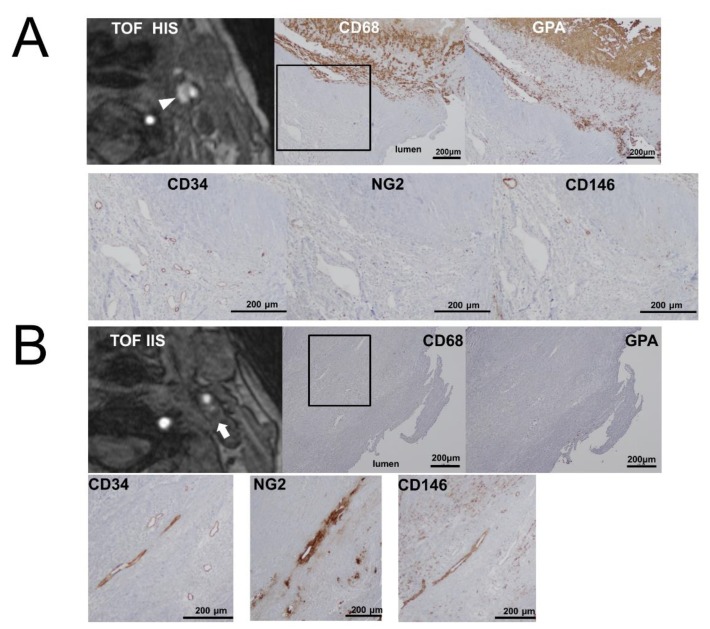
Representative cases of the TOF-HIS group (**A**) and the TOF-IIS group (**B**). Carotid plaques with high intensity (arrowhead) and iso intensity (arrow) signals on TOF-MRA. The photomicrographs of immunohistochemistry show many glycophorin A and CD68-positive cells in the TOF-HIS group. In both groups, CD34-positive neovessels are equally distributed. NG2 and CD146-positive neovessels are densely distributed in the TOF-IIS group. Square outlines indicate plaque shoulder lesions.

Overall, high glycophorin A, high CD68 expression, and low neovascularization with pericytes in the TOF-HIS group, and low glycophorin A, low CD68 expression, and high neovascularization with pericytes in the TOF-IIS group were found (Figure 3).

## 3. Discussion

The present study showed that intraplaque neovessels had higher expressions of NG2 and CD146 in iso-intensity plaques than in high-intensity plaques on TOF-MRA. Moreover, expression of CD34 in the intraplaque neovessels was not significantly different between the two groups. Additionally, a significantly higher rate of IPH in plaques with high-intensity on TOF-MRA was seen in the present study, and this finding was similar to a previous study’s finding [11]. The arterial pericytes may play a role in vascular remodeling, calcification, and intraplaque neovascularization in advanced and complicated atherosclerotic lesions [12]. There have been few reports on the role of pericytes in carotid plaque. Only two previous histopathological studies to investigate pericyte function in carotid plaque have been reported, which used anti-NG2 and CD146 antibodies as pericyte markers [7,8]. Intraplaque neovessels covered by pericytes with positive stain of NG2 and CD146 were abundant in plaques of asymptomatic patients [8] and less in plaques with severe hemorrhage [7]. The pericytes may play a role in stabilizing plaques in the carotid artery [8]. The association between radiological findings of carotid plaques and the vulnerability of intraplaque neovessels has been previously unclear. However, an association between pericytes in intraplaque neovessels and high-intensity plaque on TOF-MRA was found in the present study.

A prospective investigation of the association between TOF-MRA findings of plaques and the occurrence of ischemic stroke in patients with asymptomatic carotid stenosis has been reported. The authors found that the incidence of ischemic stroke events was significantly higher in patients with unstable plaques than in those with stable plaques on TOF-MRA [13]. Signal hyperintensity on TOF-MRA of carotid plaques suggests IPH and is associated with embolic complications during and after carotid artery stenting [14]. Therefore, the identification of an appropriate approach to stabilize unstable plaques with high-intensity TOF-MRA may contribute to the prevention of ischemic stroke events in patients with unstable carotid plaques and avoid embolic complications intra and post carotid artery stenting.

Several investigations of drug therapies targeting pericytes have been reported [15,16]. In an experimental study in animal models of ischemic stroke, out of aspirin, clopidogrel, and cilostazol, only cilostazol caused proliferation of neovessels with pericytes in the peri-infarct area [15]. Takagi et al. reported that cilostazol promoted pericyte proliferation in vitro and reduced cerebral hematoma volume in experimental animal models [16]. Therefore, the administration of cilostazol may help to activate pericytes during intraplaque angiogenesis and contribute to a decreased incidence of IPH. The occurrence of ischemic stroke has decreased due to advancements in the medical treatment of carotid stenosis, in particular for patients with asymptomatic carotid stenosis [17]. Nevertheless, the occurrence of ipsilateral ischemic stroke in asymptomatic carotid stenosis patients with IPH was reported to still be high compared to those with stable plaques [13]. Additionally, in symptomatic carotid stenosis patients undergoing medical treatment for secondary prevention, the risk of recurrent ischemic stroke was higher in patients with IPH than in those without. The relationship between the administration of antiplatelet drugs and IPH in patients with carotid artery stenosis was prospectively studied. The study showed that the occurrence of IPH was significantly elevated in patients with previous use of antiplatelet agents, and aspirin was used in 70% of the patients [18]. The optimal medical treatment for patients with carotid IPH remains unclear. To establish a treatment targeting pericytes, a treatment based on plaque features identified with non-invasive imaging examinations, such as TOF-MRA, might be beneficial.

There are some limitations of the present study. First, the sample size was relatively small, and studies with a large number of patients are warranted to obtain confirmation of the present findings. Second, the present study was cross-sectional, and thus only the relationships among factors could be shown, but the cause of IPH could not be identified. Third, the results of the present study showing that pericytes are multipotent cells that can differentiate into both mural cells and fibroblasts suggest that they may play a particular role in the healing process of IPH [19]. There was a weak relationship between macrophage infiltration and pericyte expression in intraplaque neovessels in the present study. However, an association between plaque vulnerability and CD146 expression in macrophages infiltrating human atherosclerotic plaques has been reported [20]. Therefore, the association between macrophage infiltration and pericytes in intraplaque neovessels remains unclear. Further studies are needed to establish whether pericytes are related to neovessel vulnerability.

## 4. Materials and Methods

All procedures performed in studies involving human participants were in accordance with the ethical standards of the institutional and/or national research committee and with the 1964 Helsinki declaration and its later amendments or comparable ethical standards.

This study is reported based on criteria from the STROBE (Strengthening the Reporting of Observational Study in Epidemiology) statement [21]. This study was approved by the institutional ethics board on August 2, 2019 (Approval no. 2019-05-06). Informed consent for participation in this study was obtained from all participants.

### 4.1. Patients and Study Design

In the present study, 123 consecutive patients treated with CEA at our hospitals between August 2008 and March 2016 were retrospectively reviewed. Of the 123 patients, 70 without specimens available for histopathological analysis and 7 without preoperative TOF-MRA data were excluded. A total of 46 patients with 49 carotid artery stenoses with histopathological evaluation of the plaques obtained from CEA were evaluated. The indications for CEA were symptomatic and asymptomatic patients with stenosis over 50% and 80%, respectively, according to North American Symptomatic Carotid Endarterectomy Trial (NASCET) criteria. Carotid stenosis patients with ischemic stroke events in the ipsilateral carotid artery territory within 6 months before surgery were defined as symptomatic. The presence of vascular risk factors was also recorded. Urgent CEA was defined as surgery performed within two weeks after ischemic symptoms. MRI-DWI was performed within 7 days after CEA. Restenosis after CEA was defined as >50% stenosis.

### 4.2. Preoperative Plaque Imaging and Image Analysis

A 3.0-Tesla MRI system (MAGNETOM Trio, A Tim System, Siemens AG, Erlangen, Germany or Ingenia, Philips, Netherlands) or a 1.5-Tesla MRI system (MAGNETOM Avanto, A Tim System, Siemens AG; Achieva, Philips, Netherlands; or EXCELART Vantage Atlas, Toshiba Medical Systems, Tokyo, Japan) with a 12-channel head coil was used for the examinations. The imaging parameters of the 3.0-T MRI units were as follows (3D-TOF-MRA): MAGNETOM Trio: TR/TE range = 19–20/3.1–4.9 ms, flip angle = 18–20°, field of view = 150–180 × 200, matrix size = 240–304 × 320, and slice thickness = 1.0–1.2 mm; and Ingenia: TR/TE range = 20/3.5 ms, flip angle = 18°, field of view = 120 × 200, matrix size = 152 × 304, and slice thickness = 0.8 mm. The imaging parameters of the 1.5-Tesla MRI units were as follows (3D-TOF-MRA): MAGNETOM Avanto: TR/TE range = 22–24/6.5–7.2 ms, flip angle = 20–25°, field of view = 150–180 × 200, matrix size = 192–288 × 320, and slice thickness = 1.0 mm; Achieva: TR/TE range = 18/6.9 ms, flip angle = 17°, field of view = 120 × 200, matrix size = 180 × 352, and slice thickness = 0.8 mm; and EXCELART Vantage Atlas: TR/TE range = 25/6.8 ms, flip angle = 23°, field of view = 230 × 200, matrix size = 160 × 256, and slice thickness = 1.8 mm. The scan range included the carotid bifurcation.

A certified neuroradiologist blinded to the patients’ clinical data examined the TOF-MRA axial source images of the carotid bifurcation and of the proximal internal carotid artery. Carotid plaques were defined as TOF-high if they showed a high-intensity signal >150% of the signal intensity of the adjacent neck muscle in the TOF source images [22]. Detailed methods have been previously reported [11]. On the basis of these methods, the patients were divided into TOF-HIS and TOF-IIS groups.

### 4.3. Immunohistochemical Analysis

The carotid plaque was fixed in formalin immediately after CEA and embedded in paraffin. Serial 4-μm-thick transverse sections of the largest plaque burden were prepared and then stained with hematoxylin and eosin, and the adjacent sections were stained and analyzed immunohistochemically for glycophorin A (1:800 diluted, M0819, DAKO, Glostrup, Denmark), CD68 (1:100 diluted, M0814, DAKO), CD34 (1:2000 diluted, AB81289, Abcam, Cambridge, UK), NG2 (1:400 diluted, AB5320, Merck Millipore, Billerica, MA), and CD146 (1:800 diluted, AB75769, Abcam).

Intraplaque neovessel density was counted by immunostaining with anti-CD34 antibody for endothelial cells or anti-NG2 and CD146 antibodies for pericytes [8,23]. A plaque neovessel was defined as an immunohistochemically positive tube-like formation. The number of neovessels was counted in the shoulder lesion using two sections as described previously [7]. Shoulder lesions were reportedly identified as areas between the fibrous cap and the lipid core [24]. Shoulder lesions have been reported as areas with abundant vessel formation in atheromatous plaques [25]. Micro-photographs of these sections were taken by a microscope (Olympus, Tokyo, Japan). The neovessels were counted using Image J software (1.51j8, National Institutes of Health, Bethesda, MD, USA, http://rsb.info.nih.gov/ij/download/) by two researchers who were blinded to the clinical data, and the average of these numbers was evaluated.

Macrophage infiltration was then examined. The macrophage infiltration rate in the shoulder lesion was evaluated using CD68 by one researcher (A.O.).

Finally, IPH was evaluated using glycophorin A [26]. The percentage of the glycophorin A-positive area was assessed using Image J software by a certified neurosurgeon (A.O.).

### 4.4. Statistical Analysis

Quantitative variables are expressed as means ± SD. Fisher’s exact test was used to analyze nominal data. Student’s *t*-test and the Mann–Whitney U test were used to analyze parametric and nonparametric numerical data, respectively. Spearman’s rank correlation test was used to assess correlations. The paired *t*-test was used to assess interobserver variability in the measurement of the number of intraplaque neovessels. All statistical analyses were performed using JMP Pro 11 software (SAS Institute Inc., Cary, NC, USA). A *p*-value <0.05 was considered significant.

## 5. Conclusions

A high-intensity signal in carotid plaque on TOF-MRA was associated with intraplaque hemorrhage and few pericytes in intraplaque neovessels. These findings may contribute to development of new therapeutic strategies focusing on pericytes.

## Figures and Tables

**Table 1:** Patient characteristics.

Variable	TOF-HIS (28 Lesions)	TOF-IIS (21 Lesions)	*p*-Value
Age (y)	72.8 ± 1.4	65.3 ± 2.7	0.013
Male sex (%)	28 (100)	18 (85)	NS
Rate of stenosis (%)	70.8 ± 2.7	78.7 ± 3.3	NS
Symptomatic lesions (%)	23 (82)	16 (76)	NS
Hypertension (%)	18 (64)	18 (86)	NS
Dyslipidemia (%)	21 (57)	8 (42)	NS
Diabetes mellitus (%)	12 (43)	10 (48)	NS
Coronary artery disease (%)	11 (39)	6 (28)	NS
Smoking (%)	15 (53)	13 (62)	NS
Urgent CEA (%)	8 (29)	3 (14)	NS
MRI-DWI high intensity (%)	2 (7)	3 (14)	NS
Restenosis (%)	2 (7)	1 (5)	NS

Data are presented as means ± standard deviation or numbers (%). The *p*-values for mean age and rate of stenosis are based on the Mann–Whitney U test; the other *p*-values are based on Fisher’s exact tests. NS, not significant. *p* < 0.05 was considered significant. DWI, diffusion weighted imaging.

**Table 2:** Relationship between TOF-MRA signal intensity and histopathological analysis.

Variable	TOF-HIS (28 Lesions)	TOF-IIS (21 Lesions)	*p*-Value
Area of positive stain			
Glycophorin A (%)	32.8 ± 2.7	8.3 ± 1.7	<0.0001
CD68 (%)	5.1 ± 0.7	2.2 ± 0.6	0.0039
Mean number of neovessels			
CD34	10.7 ± 1.0	10.6 ± 1.3	NS
NG2	5.0 ± 0.4	15.5 ± 2.3	<0.0001
CD146	5.9 ± 0.8	13.9 ± 1.9	<0.0001

Data are presented as means ± standard deviation or numbers (%). The *p*-values are based on the Student’s *t*-test. NS, not significant. *p* < 0.05 was considered significant.

**Table 3:** Interobserver variability in the measurement of the number of neovessels.

Variable	Observer	Mean Number of Neovessels		*p*-Value ^a^	Interobserver Variability (Paired *t*-Test *p*-Value)
		TOF HIS	TOF IIS		
CD34	Observer 1	11.2 ± 1.3	10.3 ± 1.5	0.64	0.36
	Observer 2	10.1 ± 1.2	11.0 ± 1.9	0.69	
NG2	Observer 1	4.3 ± 0.3	18.5 ± 3.0	<0.0001	0.11
	Observer 2	5.7 ± 0.7	12.6 ± 1.8	0.0003	
CD146	Observer 1	5.5 ± 1.1	10.7 ± 2.1	0.023	0.16
	Observer 2	6.2 ± 0.8	17.1 ± 2.9	0.0002	

^a^ The *p*-values are based on the Student’s *t*-test.

## Abbreviations

CD	Cluster of differentiation
CEA	Carotid endarterectomy
GPA	glycophorin A
IPH	intraplaque hemorrhage
NG2	Neuron-glial antigen 2

## References

[1] McCarthy M.J., Loftus I., Thompson M., Jones L., London N.J., Bell P.R., Naylor R., Brindle N. (1999). Angiogenesis and the atherosclerotic carotid plaque: An association between symptomatology and plaque morphology. J. Vasc. Surg..

[2] Mofidi R., Crotty T.B., McCarthy P., Sheehan S.J., Mehigan D., Keaveny T.V. (2001). Association between plaque instability, angiogenesis and symptomatic carotid occlusive disease. BJS.

[3] Hennerici M.G. (2004). The unstable plaque. Cerebrovasc. Dis..

[4] Kumamoto M., Nakashima Y., Sueishi K. (1995). Intimal neovascularization in human coronary atherosclerosis: Its origin and pathophysiological significance. Hum. Pathol..

[5] Oord S.V.D., Akkus Z., Renaud G., Bosch J.G., Van Der Steen A.F.W., Sijbrands E., Schinkel A.F. (2014). Assessment of carotid atherosclerosis, intraplaque neovascularization, and plaque ulceration using quantitative contrast-enhanced ultrasound in asymptomatic patients with diabetes mellitus. Eur. Hear. J. Cardiovasc. Imaging.

[6] Vrijenhoek J.E.P., Ruijter H.M.D., De Borst G.J., De Kleijn D.P.V., De Vries J.-P.P., Bots M.L., Van De Weg S.M., Vink A., Moll F.L., Pasterkamp G. (2013). Sex Is Associated With the Presence of Atherosclerotic Plaque Hemorrhage and Modifies the Relation Between Plaque Hemorrhage and Cardiovascular Outcome. Stroke.

[7] Tanaka T., Ogata A., Masuoka J., Mizokami T., Wakamiya T., Nakahara Y., Inoue K., Shimokawa S., Yoshioka F., Momozaki N. (2019). Possible involvement of pericytes in intraplaque hemorrhage of carotid artery stenosis. J. Neurosurg..

[8] Davaine J.-M., Quillard T., Brion R., Laperine O., Guyomarch B., Merlini T., Chatelais M., Guilbaud F., Brennan M.Á., Charrier C. (2014). Osteoprotegerin, Pericytes and Bone-Like Vascular Calcification Are Associated with Carotid Plaque Stability. PLoS ONE.

[9] Armulik A., Genové G., Betsholtz C., Keller A. (2011). Pericytes: Developmental, Physiological, and Pathological Perspectives, Problems, and Promises. Dev. Cell.

[10] Takaya N., Yuan C., Chu B., Saam T., Polissar N.L., Jarvik G.P., Isaac C., McDonough J., Natiello C., Small R. (2005). Presence of Intraplaque Hemorrhage Stimulates Progression of Carotid Atherosclerotic Plaques. Circulation.

[11] Ogata A., Kawashima M., Wakamiya T., Nishihara M., Masuoka J., Nakahara Y., Ebashi R., Inoue K., Takase Y., Irie H. (2016). Carotid artery stenosis with a high-intensity signal plaque on time-of-flight magnetic resonance angiography and association with evidence of intraplaque hypoxia. J. Neurosurg..

[12] Orekhov A.N., Bobryshev Y.V., Chistiakov D.A. (2014). The complexity of cell composition of the intima of large arteries: Focus on pericyte-like cells. Cardiovasc. Res..

[13] Esposito-Bauer L., Saam T., Ghodrati I., Pelisek J., Heider P., Bauer M., Wolf P., Bockelbrink A., Feurer R., Sepp D. (2013). MRI Plaque Imaging Detects Carotid Plaques with a High Risk for Future Cerebrovascular Events in Asymptomatic Patients. PLoS ONE.

[14] Yoshimura S., Yamada K., Kawasaki M., Asano T., Kanematsu M., Takamatsu M., Hara A., Iwama T. (2011). High-Intensity Signal on Time-of-Flight Magnetic Resonance Angiography Indicates Carotid Plaques at High Risk for Cerebral Embolism During Stenting. Stroke.

[15] Omote Y., Deguchi K., Kono S., Liu N., Liu W., Kurata T., Yamashita T., Ikeda Y., Abe K. (2013). Neurovascular protection of cilostazol in stroke-prone spontaneous hypertensive rats associated with angiogenesis and pericyte proliferation. J. Neurosci. Res..

[16] Takagi T., Imai T., Mishiro K., Ishisaka M., Tsujimoto M., Ito H., Nagashima K., Matsukawa H., Tsuruma K., Shimazawa M. (2016). Cilostazol ameliorates collagenase-induced cerebral hemorrhage by protecting the blood–brain barrier. Br. J. Pharmacol..

[17] Abbott A. (2009). Medical (Nonsurgical) Intervention Alone Is Now Best for Prevention of Stroke Associated With Asymptomatic Severe Carotid Stenosis. Stroke.

[18] Liem M.I., Schreuder F.H., Van Dijk A.C., De Rotte A.A.J., Truijman M.T., Daemen M.J., Van Der Steen A.F., Hendrikse J., Nederveen A.J., Van Der Lugt A. (2015). Use of Antiplatelet Agents Is Associated With Intraplaque Hemorrhage on Carotid Magnetic Resonance Imaging. Stroke.

[19] Makihara N., Arimura K., Ago T., Tachibana M., Nishimura A., Nakamura K., Matsuo R., Wakisaka Y., Kuroda J., Sugimori H. (2015). Involvement of platelet-derived growth factor receptor beta in fibrosis through extracellular matrix protein production after ischemic stroke. Exp. Neurol..

[20] Qian Y.-N., Luo Y.-T., Duan H., Feng L.-Q., Bi Q., Wang Y., Yan X.-Y. (2014). Adhesion Molecule CD146 and its Soluble Form Correlate Well with Carotid Atherosclerosis and Plaque Instability. CNS Neurosci. Ther..

[21] Von Elm E., Altman U.G., Egger M., Pocock S.J., Gøtzsche P.C., Vandenbroucke J.P. (2007). The Strengthening the Reporting of Observational Studies in Epidemiology (STROBE) Statement: Guidelines for Reporting Observational Studies. PLoS Med..

[22] Altaf N., MacSweeney S.T., Gladman J.R.F., Auer D.P. (2007). Carotid Intraplaque Hemorrhage Predicts Recurrent Symptoms in Patients with High-Grade Carotid Stenosis. Stroke.

[23] Hall A.P. (2006). Review of the Pericyte during Angiogenesis and its Role in Cancer and Diabetic Retinopathy. Toxicol. Pathol..

[24] Olson F.J., Strömberg S., Hjelmgren O., Kjelldahl J., Fagerberg B., Bergström G. (2011). Increased vascularization of shoulder regions of carotid atherosclerotic plaques from patients with diabetes. J. Vasc. Surg..

[25] Jeziorska M., Woolley D.E. (1999). Local neovascularization and cellular composition within vulnerable regions of atherosclerotic plaques of human carotid arteries. J. Pathol..

[26] Kolodgie F.D., Gold H.K., Burke A.P., Fowler D., Kruth H.S., Weber D.K., Farb A., Guerrero L., Hayase M., Kutys R. (2003). Intraplaque Hemorrhage and Progression of Coronary Atheroma. N. Engl. J. Med..

